# Integrated disease model considering mutation-induced infection waves with COVID-19 cases

**DOI:** 10.1371/journal.pone.0341667

**Published:** 2026-03-06

**Authors:** Seungho Baek, Haneol Cho, SangChul Lee, Myeongsu Yoo, Donghyok Kwon, KyuHwan Lee, Yeonju Kim, Chansoo Kim

**Affiliations:** 1 AI⋅Information⋅Reasoning (AI/R) Laboratory, Computational Science Center, Korea Institute of Science and Technology, Seoul, Republic of Korea; 2 AI-Robot Department, University of Science and Technology, Daejeon, Republic of Korea; 3 Department of Computer Science, Sejong University, Seoul, Republic of Korea; 4 Korea Disease Control Agency, Cheongju-si, Republic of Korea; 5 Department of Medicine Science, Inha University College of Medicine, Incheon, Republic of Korea; Centers for Disease Control and Prevention, UNITED STATES OF AMERICA

## Abstract

COVID-19, an unprecedented global pandemic, has caused successive waves that pose unique challenges to public health and epidemiological research. Traditional Susceptible–Infected–Recovered (SIR) models often struggle to capture these complex dynamics, especially given that the virus has spawned multiple sub-variants. To tackle these challenges, we adopt an empirical modeling approach by integrating real-world data from *Our World in Data* (daily confirmed COVID-19 cases) and *GISAID* (variant prevalence) into a newly proposed integrated model. Specifically, we sum multiple sigmoidal (logistic) curves, each representing the cumulative infections of a distinct dominant variant, and recalibrate the model whenever a new variant emerges. By incorporating variant-specific parameters, our framework effectively captures the biological and epidemiological characteristics of COVID-19 in a dynamic, data-driven manner. Most importantly, we employ the Pruned Exact Linear Time (PELT) algorithm to provide rigorous mathematical justification for when separate variant models can be legitimately summed: specifically, when variant dominance reaches approximately 50%. This establishes the theoretical foundation that separate logistic models can be additively combined under specific dominance conditions. We evaluate our model using Mean Absolute Percentage Error (MAPE), Root Mean Square Error (RMSE), and Mean Absolute Error (MAE). Our empirical findings confirm that integrating dominant variants is associated with markedly improved accuracy compared to a single-strain approach, based on data from fourteen countries (including South Korea, the United States, and the United Kingdom) and global aggregates. We also provide theoretical motivation for approximating SIR dynamics via logistic functions and discuss how this integrated framework can be refined to enhance predictive performance. In doing so, we demonstrate the feasibility and advantages of iteratively updating epidemiological models in response to emerging variants, thereby offering actionable insights for ongoing and future pandemic management.

## Introduction

Since the outbreak of the novel coronavirus disease (COVID-19), which was first reported in Wuhan, China, in December 2019 [1–3], a vast array of mathematical and computational models have been developed to understand and predict the pandemic’s course. The canonical Susceptible–Infected–Recovered (SIR) model, described in Eq (1), served as a cornerstone in early analyses, offering insight into the basic dynamics of infectious diseases [4–8].

$$\frac{dS}{dt}=-\beta SI,\;\frac{dI}{dt}=\beta SI-\gamma I,\;\frac{dR}{dt}=\gamma I,$$ (1)

**Fig 1:**
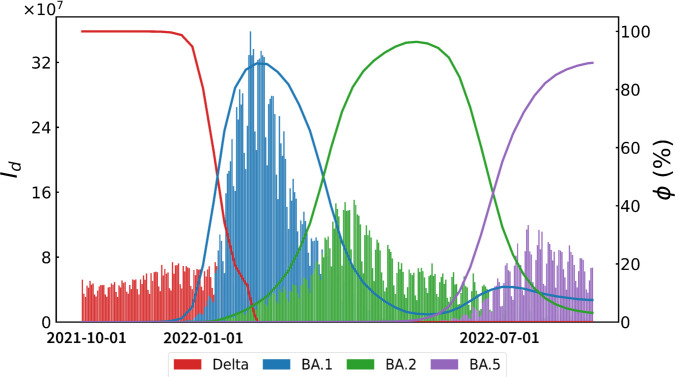
Worldwide confirmed cases and variant ratios. *I*_*d*_ (left) is the global confirmed COVID-19 cases from October 2021 to August 2022; *ϕ* (right) represents the variant ratio over the same period, corresponding to the timeline.

In the above formulation, *S*, *I*, and *R* represent the susceptible, infected, and recovered (or removed) populations, respectively; *β* is the transmission rate, and *γ* is the recovery rate. Various extensions to the SIR framework have been proposed to incorporate practical elements such as social distancing [4–7,9–11] and vaccination strategies [12–14], and these models have proved valuable for preparedness and risk management [15–18]. Despite these advances, many early predictions underestimated the pandemic’s duration, failing to account for ongoing waves of infection driven by newly emerging variants [19,20]. In particular, the Omicron variant exhibited a remarkable transmission rate, approximately threefold higher than previous strains, even among fully vaccinated populations [21–23], and drastically reduced vaccine effectiveness [14,24–26]. The repeated mutation of the SARS-CoV-2 virus has thus challenged the assumption of a single dominant strain, revealing limitations of traditional single-strain SIR models that do not explicitly incorporate variant dynamics [27–34]. Fig 1 shows how each distinct wave from late 2021 to mid 2022 closely aligns with the emergence of a specific variant (Delta, BA.1, BA.2, BA.5, and so forth).

The emergence of virus mutations plays a significant role in driving new waves of pandemics [35,36]. As shown in Fig 1, from October 2021 to August 2022, four distinct waves were observed, each moving in tandem with the emergence of a different variant of the virus. This means that one virus can be seen as a series of viral waves driven by successive mutations. Epidemiological models that are predicated on a single strain or that do not include viral mutations may not adequately express the dynamics of disease propagation. Therefore, as viruses mutate over time and spread rapidly, the predictability of the SIR model may decrease [27,37], since traditional SIR models do not take into account the effects of new variants [28–30,38]. In response to changes in the pandemic, many researchers expanded their models to a dynamic SIR model that accounted for vaccine uptake and effectiveness and new variants in estimating cases, hospitalization, deaths, and the number of necessary beds [31,39].

In recent research, the subsequent waves of COVID-19 were represented through a combination of five wavelets [40], demonstrating superior results compared to predictions from the ARIMA model. Research by Kaxiras [29] added a 14-day cycle S-I-R model to represent sub-variants of COVID-19. However, these precedents lacked evidence for representing sub-variants with a single SIR or wavelet. Vasconcelos’s research [41] modeled the long-term waves of COVID-19 by summing single SIR models for each sub-variant and utilized time parameters to accurately represent peaks, though concerns of overfitting arose due to the SIR model having six parameters. Finally, Gerardo’s [28] study examined the modeling of sub-variants for Ebola and SARS infections but showed potential overfitting and insufficient biological evidence for simply adding peaks of various SIR models. In addition, Lazebnik [42] proposed a generalized multi-strain SIR framework that highlights theoretical challenges in modeling more than two competing pathogens. While analytically interesting, their model does not utilize real-world prevalence data, which limits its practical applicability to observed variant transitions. Here, we consider the question: if a bimodal approach is superior to a unimodal approach, why not fit all sub-variants of Omicron using a multimodal model? As mentioned, there are over 50 reported sub-variants of the Omicron virus. However, this approach has been previously explored, and fitting all these variants into a single model risks creating an unproductive overfitted model. In this research, we demonstrate through calculations that a model incorporating only two dominant variants can sufficiently describe the actual data. In this study, we approximate each variant’s SIR wave by a logistic function, and sum multiple logistic curves to capture successive waves. We present a model that effectively captures the ongoing waves by incorporating the characteristics of mutant viruses. Additionally, we establish the biological basis for this model, providing an interpretation of the continuous waves. This approach is expected to enable more accurate and realistic predictions of infectious disease spread and support the development of effective response strategies.

## Materials and methods

### Heuristic model

In this study, we employ the following logistic form as the foundational basis for modeling infectious diseases:

$$I_{c}(t)=\frac{a}{1+\exp\{\;c\;(t-b)\}}\;,$$ (2)

where *I*_*c*_(*t*) denotes the cumulative number of confirmed cases at time *t*. The parameter *a* represents the final epidemic size (i.e., the upper bound of the cumulative infected), *b* is the inflection point at which the epidemic growth is most rapid, and *c* is a rate parameter that governs the speed of infection spread. We fit Eq (2) to the observed cumulative cases for each dominant variant period, using an ordinary least-squares approach. This logistic equation can also be viewed as an approximation to the cumulative infections derived from a classical SIR framework under certain simplifying assumptions. Specifically, if we define $I_{c}^{\prime }(t)$ as the rate of new infections, then under the approximation S(t)≈N (where *N* is the total population) we have


$$I_{c}^{\prime }(t)\;=\;\beta \frac{S(t)\;I(t)}{N}\;\approx \;\beta \;I(t),$$

which, upon integration, yields a logistic-type solution for *I*_*c*_(*t*) [28,29]. Hence, *a*, *b*, and *c* can be interpreted, respectively, as the eventual maximum of cumulative infections, the time at which growth transitions (inflection point), and the overall infection growth rate.

However, real-world COVID-19 data often exhibit multiple waves due to the emergence of distinct variants over time. To capture such successive waves, we integrate two (or more) logistic terms, each corresponding to a dominant variant. For example, if two dominant variants drive consecutive waves, an integrated model can be written as:

$$I_{c}(t)\;=\;\frac{a_{1}}{1+\exp\{c_{1}(t-b_{1})\}}\;+\;\frac{a_{2}}{1+\exp\{c_{2}(t-b_{2})\}}.$$ (3)

Each logistic term models the cumulative infections of a specific variant with its own parameters *a*_*i*_, *b*_*i*_, and *c*_*i*_. In the sections that follow, we show how this logistic function is fitted to the COVID-19 data and how each parameter (*a*, *b*, *c*) reflects the epidemic characteristics. We then extend the model by incorporating additional logistic terms to account for new dominant variants over time.

### Data extraction and selection criteria

The heuristic model is based on daily COVID-19 confirmed case data from the COVID-19 Data Repository by the Center for Systems Science and Engineering (CSSE) at Johns Hopkins University [43] and Our World in Data (OWiD) [44]. The study period spans from October 2021 to September 2022, encompassing four major variant transitions (Delta, BA.1, BA.2, BA.5). This period was selected to ensure sufficient temporal coverage for model validation while maintaining data reliability before the global scale-down of COVID-19 surveillance systems [45,46].

The data of the viral mutations in confirmed cases were obtained from GISAID [47], providing data that details the distribution of mutated viruses in various countries.

We selected fourteen countries (United States, United Kingdom, Japan, Australia, Canada, France, Israel, South Korea, Chile, Denmark, Germany, South Africa, Singapore) and global aggregates based on two criteria: (1) availability of consistent genomic sequencing data from GISAID, and (2) reliability of daily case reporting. Countries with known data quality issues (e.g., China) were excluded from the analysis.

Data were smoothed using a 7-day moving average to mitigate reporting fluctuations (e.g., weekend effects). For countries with weekly reporting schedules (France, and Canada and Australia after June 2022), linear interpolation was applied to estimate daily values before smoothing.

### Statistical analysis

Model parameters (*a*, *b*, *c*) in Eq (2) were estimated using nonlinear least squares optimization (Levenberg–Marquardt algorithm) implemented in Python’s SciPy library (version 1.7.3). Initial parameter values were set heuristically: the epidemic size *a* was initialized as the maximum cumulative cases in the observation window, the inflection point *b* as the midpoint of the observation period, and the growth rate *c* as 0.1.

To rigorously evaluate model performance, we employed three complementary error metrics: Mean Absolute Percentage Error (MAPE, Eq 5), Root Mean Square Error (RMSE), and Mean Absolute Error (MAE):

$$\text{RMSE}=\sqrt{\frac{1}{n}\sum_{i=1}^{n}(y_{i}-{\hat{y}}_{i}{)}^{2}},\;\text{MAE}=\frac{1}{n}\sum_{i=1}^{n}|y_{i}-{\hat{y}}_{i}|$$ (4)

where *y*_*i*_ denotes observed values, ${\hat{y}}_{i}$ represents model estimates, and *n* is the number of observations. MAPE provides scale-independent comparison across countries, RMSE emphasizes larger deviations during peak periods, and MAE offers interpretable absolute error in case counts.

### Mean absolute percentage error

In our study, we employed the Mean Absolute Percentage Error (MAPE) to evaluate the accuracy of our integrated model. MAPE is a standard measure of forecast accuracy in various analytical fields, quantifying the average absolute percentage difference between observed values and those predicted by the model. This measure is particularly beneficial for its interpretability as it presents errors as a percentage. The formula for MAPE is expressed as follows:

$$\text{MAPE}=\left(\frac{1}{n}\sum_{i=1}^{n}\left|\frac{y_{i}-{\hat{y}}_{i}}{y_{i}}\right|\right)\times 100\%$$ (5)

where *y*_*i*_ denotes the actual data values, ${\hat{y}}_{i}$ represents the estimates provided by the integrated model, and *n* is the total number of observations. This metric allows for a straightforward comparison of model performance across different datasets or modeling approaches.

### Mathematical justification for additive model combination

The additive combination of multiple logistic models requires theoretical justification, particularly regarding the timing of model integration. Let $I_{\text{total}}(t)$ denote the total cumulative incidence. When a single variant *V*_1_ dominates ($\phi_{V_{1}}\approx 1$), the dynamics are well-approximated by $I_{\text{total}}(t)\approx I_{V_{1}}(t)$. As a new variant *V*_2_ emerges and gains prevalence, the total incidence transitions to a superposition:

$$I_{\text{total}}(t)=I_{V_{1}}(t)+I_{V_{2}}(t)$$ (6)

The critical question is: what prevalence threshold *ϕ* does this additive formulation become necessary?

Our empirical analysis using the PELT algorithm (Fig 3) identifies this change point at approximately ϕ≈50% (ranging from 50% for BA.1 to 56% for BA.5). This aligns with the theoretical intuition that when a new variant comprises the majority of incident cases (ϕ>50%), the epidemiological dynamics are no longer adequately captured by the declining wave of the previous variant alone; instead, the superposition of both variant-specific curves is required.

**Fig 2:**
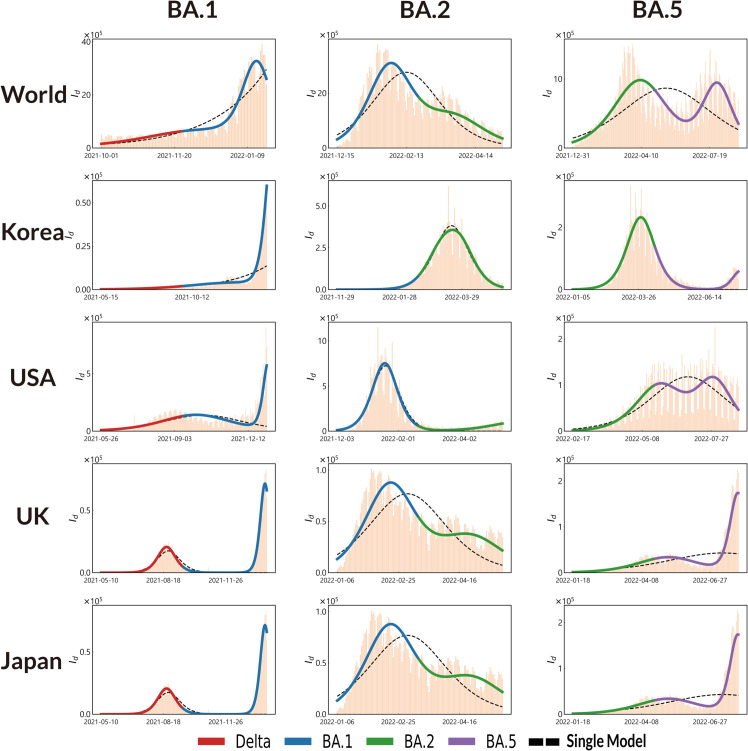
Integrated and single models for variants in Worldwide, South Korea, the US, the UK and Japan. Each figure’s bar chart represents daily confirmed cases. The black dashed line indicates the estimates from the single model, and the colored segments represent the estimates from the integrated model, which are the sum of the two variant viruses.

**Fig 3:**
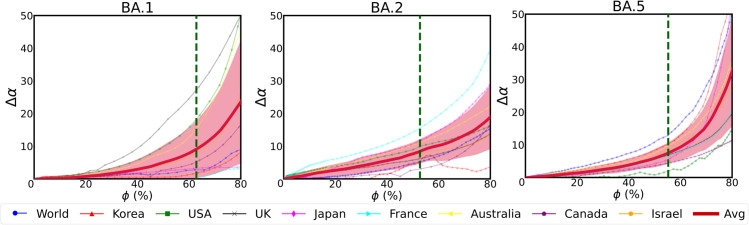
Model application limit point. The graph displays the difference in error values between the two models for each country on the y-axis, represented as Δα. The prevalence of new variant strains is represented by *ϕ* on the x-axis. As a new variant emerges and triggers a new wave, the single model increasingly fails to fit the data. This leads to a sharp rise in its error compared to the integrated model. The average Δα across the nine cases is represented by a bold red line. The green vertical line indicates the abrupt change point in the time series data (the red line), as detected by the PELT algorithm.

This additive formulation assumes that infections from different variants can be treated as largely independent during their respective dominance periods. During the early-to-mid pandemic phase studied (October 2021, September 2022), each major variant exhibited significant antigenic differences from its predecessors, and population immunity remained limited, supporting this independence assumption. The implications and limitations of assumption are discussed in the Limitations section.

### PELT algorithm

The PELT (Pruned Exact Linear Time) algorithm was employed to detect changes in the accuracy of our model. PELT is an advanced change point detection method that identifies shifts in statistical properties within a dataset. The algorithm optimizes the detection process by minimizing a cost function, which combines a measure of fit (such as the residual sum of squares) and a penalty term for the number of change points. The cost function is given by:

$$C(t)=\sum_{i=1}^{t}(y_{i}-{\hat{y}}_{i}{)}^{2}+\beta K$$ (7)

where *C*(*t*) is the cost up to time *t*, *y*_*i*_ are the observed values, ${\hat{y}}_{i}$ are the predicted values, *β* is a penalty constant determined via the Bayesian Information Criterion (BIC), and *K* is the number of change points. The algorithm prunes unnecessary calculations to achieve linear time complexity, ensuring efficiency even with large datasets. In our study, we applied PELT to identify significant change points in the accuracy of the model over time. By analyzing the MAPE values before and after the detected change points, we demonstrated that the enhanced model, incorporating the improvements, significantly outperformed the original model post-change point.

## Results

### Comparative evaluation of integrated and single models

Our integrated model is designed to capture the unique epidemiological properties of each variant by fitting separate SIR models (approximated by a logistic function) to the data corresponding to during the dominance period of each variant. These individual models are then combined to create a comprehensive representation of the pandemic’s trajectory over time. To evaluate the performance of our integrated model, we conducted a comprehensive analysis using data from fourteen cases: the United States, the United Kingdom, Japan, Australia, Canada, France, Israel, South Korea, Chile, Denmark, Germany, South Africa, Singapore and global data. Accuracy was measured using the Mean Absolute Percentage Error (MAPE), a widely used metric for assessing the accuracy of models against non-negative actual time-series data [48]. Additionally, RMSE and MAE were computed to provide complementary perspectives on model accuracy (S1 Table). MAPE calculates the error by measuring the deviation between model estimates and the actual data. We focused on the four most prevalent Omicron subvariants: Delta, BA.1, BA.2, and BA.5. In all cases, our integrated model consistently outperformed the single SIR model in terms of its ability to accurately capture the observed dynamics of the pandemic. To illustrate these accuracy improvements, we present examples in Fig 2 that showcase diverse epidemiological contexts. This improved performance was evident in the model’s ability to better fit the data during periods of transition between dominant variants, as well as in its overall accuracy throughout the study period.

The error reduction rates shown in Table 1 exhibit varying levels of improvement across different regions as new variants emerge. Globally, the reduction rates increased steadily from 12.89%p (Delta–BA.1) to 19.84%p (BA.1–BA.2) and 25.46%p (BA.2–BA.5). In Korea, notable improvement was observed between BA.2 and BA.5 (42.24%p), whereas the reduction was more modest (3.51%p) between BA.1 and BA.2, reflecting the temporal overlap of these variants within a two-week interval. The USA showed balanced improvements across all variant transitions, ranging from 22.88%p to 25.49%p. The UK exhibited significant gains, especially during the later transitions (43.91%p for BA.2–BA.5). Japan displayed strong performance in both early (Delta–BA.1: 49.15%p) and later transitions (BA.2–BA.5: 49.23%p). In France, the smallest reduction (5.03%p) was noted initially, but larger gains (62.65%p) appeared from BA.2 to BA.5. Australia similarly demonstrated considerable improvement (49.29%p) for the BA.2–BA.5 transition. Canada recorded a higher error reduction (31.44%p) for the BA.1–BA.2 transition, while Israel displayed its largest improvement (58.33%p) in the same transition. Overall, these findings underscore the consistent benefit of incorporating emerging variants into the model, even though the magnitude of improvement varies according to local epidemiological and demographic factors. Additionally, we extended our analysis to five more countries—Chile, Denmark, Germany, South Africa, and Singapore—and present those results in Supporting Information. These supplementary findings confirm similar patterns of improvement, further supporting the robustness of our integrated model.

**Table 1:** Error reduction rates (%p) for each case (MAPE). Additional error metrics (RMSE, MAE) are presented in S1 Table.

Case	Delta-BA.1	BA.1-BA.2	BA.2-BA.5
World	12.89	19.84	25.46
Korea (kr)	21.41	3.51	42.24
USA (us)	25.49	24.36	22.88
UK (uk)	31.34	35.94	43.91
Japan (jp)	49.15	18.34	49.23
France (fr)	5.03	30.64	62.65
Australia (au)	36.56	15.70	49.29
Canada (ca)	11.45	31.44	22.92
Israel (is)	35.52	58.33	34.96

### Model fit assessments and applying criteria by PELT algorithm

We have computed the model’s error using MAPE for three cases: when the BA.1, BA.2, and BA.5 variants emerged. MAPE measures the mean percentage error between the fitted and observed values, indicating how much the model deviates from the actual data. As a new variant emerges and triggers a new wave, the error of the single model increasingly grows. This leads to a sharp rise in its error compared to the integrated model. In Fig 3, we represent the difference in MAPE values between the integrated model and the single model as Δα. As the prevalence of a new variant *ϕ* increases, the rising Δα value indicates that the error of the single model increases relative to that of the integrated model. We applied the PELT algorithm to the red line, which represents the average of all Δα values, to identify the point where the error of the single model increases most sharply. Typically, fitting single model after approximately 50% for BA.1, 54% for BA.2, and 56% for BA.5 resulted in a sharp increase in error. Therefore, when the prevalence of a new variant exceeds about 50% (When it becomes dominant), it is essential to use a model that accounts for the variant rather than relying on a standard SIR model.

### Sensitivity analysis

To assess the robustness of our findings, we conducted sensitivity analyses on key modeling parameters.

**Dominance threshold.** Based on the error improvement patterns shown in Fig 3, we examined model performance across different prevalence thresholds for integrating additional variant components. The integrated model consistently outperforms the single model across thresholds from approximately 40% to 60%. The PELT algorithm identified thresholds around 50% (BA.1), 54% (BA.2), and 56% (BA.5), all falling within this optimal range, confirming that our empirically-derived criterion is robust to reasonable variations in threshold selection.

**Error metrics.** The superiority of the integrated model was confirmed across all three metrics (MAPE, RMSE, MAE), with consistent patterns of improvement observed both globally and across individual countries (S1 Table). This consistency across metrics with different mathematical properties strengthens confidence in the robustness of our findings.

## Discussion

### Summary

We have demonstrated that emerging and dominant variants are significantly associated with the epidemiological waves of COVID-19. Incorporating these variants into a single model markedly enhances the performance relative to a single-strain SIR approach. Using data from various countries (e.g., South Korea, the United Kingdom), we confirmed that variant-specific modeling captures the real-world trajectory more accurately. Moreover, we found that a new wave generally appears every 2–3 months when a new variant emerges, suggesting that policymakers can utilize this interval to design and implement public health strategies.

### Country-specific case analysis

In Fig 2, while variations exist across different regions, an important observation is that Omicron sublineages often manifest a new peak every two months. This timing allows local authorities to respond proactively. Whenever major variants (Delta, BA.1, BA.2, BA.5, etc.) were dominant, our integrated model provided superior fits compared to a single logistic curve. Significant improvements occurred when the timing of variant emergence was clearly distinct, producing bimodal or multimodal distributions in the data. For example, the worldwide shift from BA.2 to BA.5, as well as the United Kingdom’s shifts from BA.1 to BA.2 and from BA.2 to BA.5, yielded error reductions exceeding 40%. Conversely, in South Korea, where the development periods of BA.1 and BA.2 overlapped within a two-week interval, the wave resembled that of a single dominant strain, resulting in only modest improvement (a 3.51 percentage-point reduction). Additionally, there are special cases like South Korea’s shift from Delta to BA.1 and Japan’s shift from Delta to BA.1, where stringent public health measures initially suppressed the pandemic, delaying the first peak until BA.1 eventually spread widely. Despite this suppression, our integrated model still captured the subsequent sharp increase, confirming its robustness against such irregular wave patterns. From these findings, we conclude that clearly separated variant waves yield the greatest enhancement in modeling accuracy. Based on Fig 3, focusing on new variants once they reach about 50% prevalence is sufficient to reflect real data without overfitting. Minor sub-variants only marginally affect the model estimates and do not undermine the approach.

In the realm of infectious disease modeling, conventional models such as SIR often encounter challenges when applied to pathogens like SARS-CoV-2, which exhibit frequent mutations and recurrent waves. While combining single heuristic models may appear straightforward, such an approach can risk overfitting and lacks solid biological justification. Previous work has introduced parameters for reinfection [49] or viewed recurrent waves as a train of smaller wavelets [29]. Although these attempts capture some nuances, our model explicitly tracks dominant variants, which allows for improved interpretability and offers enhanced predictive performance in contexts where variant-driven dynamics are prominent. Compared to Lazebnik’s multi-strain SIR framework [42], which provides theoretical insights but lacks real-world prevalence data integration, our approach directly incorporates observed variant frequencies from GISAID. Similarly, while Vasconcelos’s summed SIR approach [41] risks overfitting with six parameters per wave, our logistic approximation achieves comparable flexibility with only three parameters per variant, reducing overfitting risk while maintaining biological interpretability. A comprehensive quantitative comparison with SEIR models and machine learning methods remains an important direction for future research.

### Future improvements

Our analysis deals with a period from October 2021 to September 2022. This cutoff reflects data availability constraints rather than methodological limitations. Following the decline of the pandemic emergency phase, global COVID-19 surveillance systems were substantially reduced: the European Centre for Disease Prevention and Control (ECDC) discontinued non-EU/EEA data collection in June 2022 [45]; the World Health Organization declared in August 2023 that daily reporting was no longer required [46]; and the U.S. Centers for Disease Control and Prevention transitioned multiple surveillance systems following the end of the public health emergency in May 2023 [50]. These changes resulted in inconsistent data quality after mid-2022, precluding reliable model extension. Nevertheless, our study period captures four distinct variant-driven waves, providing sufficient temporal coverage for demonstrating the utility of our integrated approach.

Our integrated model can be fitted quickly to data from diverse regions and can be extended to account for additional factors such as super-spreader events, policy relaxations, or changes in vaccine efficacy. In subsequent studies, extending this methodology to alternative models could enhance forecasting reliability and offer deeper insights into multi-variant disease dynamics under various real-world scenarios [51–53]. Additionally, incorporating explicit vaccination dynamics, cross-immunity parameters, and real-time variant surveillance data could improve the model’s applicability to endemic-phase COVID-19 and other respiratory pathogens.

## Conclusion

In this study, we demonstrated that incorporating variant-specific data into an integrated SIR model significantly enhances its performance in representing the dynamics of COVID-19 outbreaks. Our analysis confirms that the emergence and dominance of new variants are closely associated with the epidemiological waves observed during the pandemic. The integrated model effectively captures these dynamics, especially when the timing of variant emergence is distinct, resulting in improved accuracy over traditional single-strain models. Our findings highlight that the integrated model excels when the periods of variant dominance are temporally separated, allowing for clear bimodal distributions. This approach reduces model error by over 40% in cases with non-overlapping variant emergence, underscoring the importance of accurately modeling distinct waves of infections. Conversely, in scenarios where variants emerge and overlap in time, such as in South Korea, the model still shows improvement, albeit modest, demonstrating its robustness even under less ideal conditions. Comparisons with prior works suggest that indiscriminate aggregation of multiple SIR curves (or multi-wave heuristics) without considering biological evidence can lead to inaccuracies. Our integrated approach, by focusing on dominant variants and avoiding unnecessary complexity, addresses these limitations and provides a more biologically grounded framework. Moreover, the model’s adaptability and ease of application to various regions and data sets make it a valuable tool for ongoing and future pandemic response strategies. Overall, this study contributes to the evolving landscape of infectious disease modeling by offering a more precise and adaptable approach to understanding and estimating the spread of COVID-19 and its variants. The integrated model not only improves our comprehension of the pandemic’s trajectory but also supports the development of targeted and effective public health interventions. Future work should focus on expanding the model’s application to a wider range of scenarios and incorporating additional data sources to further refine its predictive capabilities.

## Supporting information

S1 Text Integrated and Single Models for Variants in Australia, Canada, France and Israel. Each figure’s bar chart represents daily confirmed cases. The black dashed line indicates the estimates from the single model, and the colored segments represent the estimates from the integrated model, which are the sum of the two variant viruses.
(PDF)

S2 Text Integrated and Single Models for Variants in Chile, Denmark, Germany, South Africa and Singapore. Each figure’s bar chart represents daily confirmed cases. The black dashed line indicates the estimates from the single model, and the colored segments represent the estimates from the integrated model, which are the sum of the two variant viruses.
(PDF)

S3 Text Residual time series for single and integrated models in global total and the United States. Each panel shows residuals (observed minus fitted daily cases) for the single logistic model (dashed lines) and the integrated model (solid lines) across three variant transitions (Delta–BA.1, BA.1–BA.2, and BA.2–BA.5). Columns correspond to variant transitions and rows correspond to regions (World and USA).
(PDF)

S4 Text Comparison of error metrics across countries. MAPE, RMSE, and MAE values for the single model and integrated model across all study countries and variant transitions.
(PDF)

S5 Text Error reduction rates for additional countries. MAPE improvement (%p) for Chile, Denmark, Germany, South Africa, and Singapore across three major variant transitions (Delta–BA.1, BA.1–BA.2, BA.2–BA.5).
(PDF)

S6 Text Algorithm of Detection of structural transition in accuracy improvement. Detailed algorithm for applying PELT to averaged accuracy improvement curves.
(PDF)
