# Supplementary material for: Integrated disease model considering mutation-induced infection waves with COVID-19 cases
Source: PLoS One. 2026 Mar 6;21(3):e0341667. doi: 10.1371/journal.pone.0341667 (PMC12965675; doi:10.1371/journal.pone.0341667)
Supplement: S2 Text — Each figure’s bar chart represents daily confirmed cases. The black dashed line indicates the estimates from the single model, and the colored segments represent the estimates from the integrated model, which are the sum of the two variant viruses. (PDF) [file pone.0341667.s002.pdf]

Supporting Information

Integrated Disease Model Considering Mutation Induced Infection Waves with COVID-19 Cases

Seungho Baek *et al.*
Corresponding Author: Chansoo Kim, eau@ust.ac.kr.

S2. Integrated and Single Models for Variants in Chile, Denmark, Germany, South Africa and Singapore

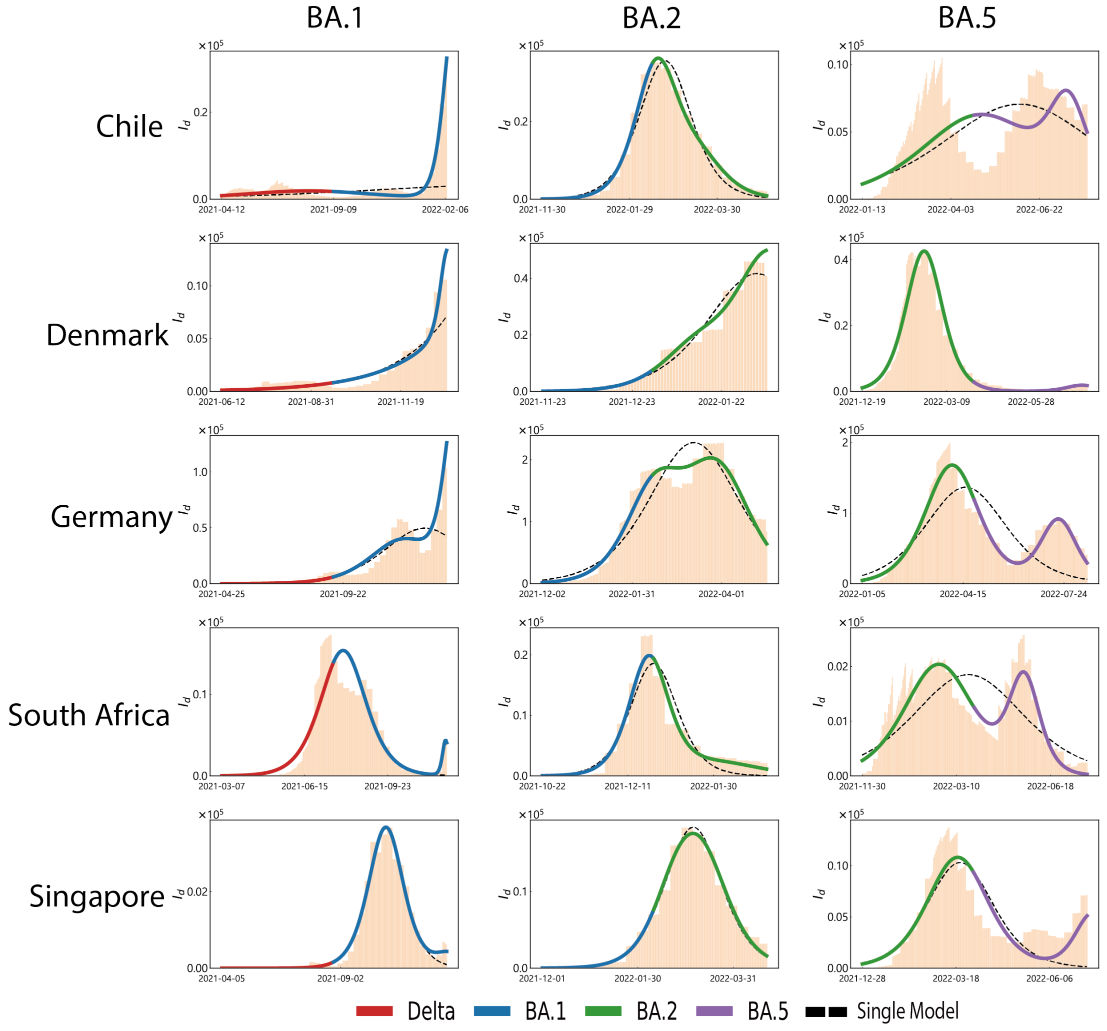

S2: Integrated and Single Models for Variants in Chile, Denmark, Germany, South Africa and Singapore. Each figure's bar chart represents daily confirmed cases. The black dashed line indicates the estimates from the single model, and the colored segments represent the estimates from the integrated model, which are the sum of the two variant viruses.
