# Supplementary material for: Integrated disease model considering mutation-induced infection waves with COVID-19 cases
Source: PLoS One. 2026 Mar 6;21(3):e0341667. doi: 10.1371/journal.pone.0341667 (PMC12965675; doi:10.1371/journal.pone.0341667)
Supplement: S3 Text — Each panel shows residuals (observed minus fitted daily cases) for the single logistic model (dashed lines) and the integrated model (solid lines) across three variant transitions (Delta–BA.1, BA.1–BA.2, and BA.2–BA.5). Columns correspond to variant transitions and rows correspond to regions (World and USA). (PDF) [file pone.0341667.s003.pdf]

Supporting Information

Integrated Disease Model Considering Mutation Induced Infection Waves with COVID-19 Cases

Seungho Baek *et al.*
Corresponding Author: Chansoo Kim, eau@ust.ac.kr.

S3. Residual time series for single and integrated models in global total and the United States

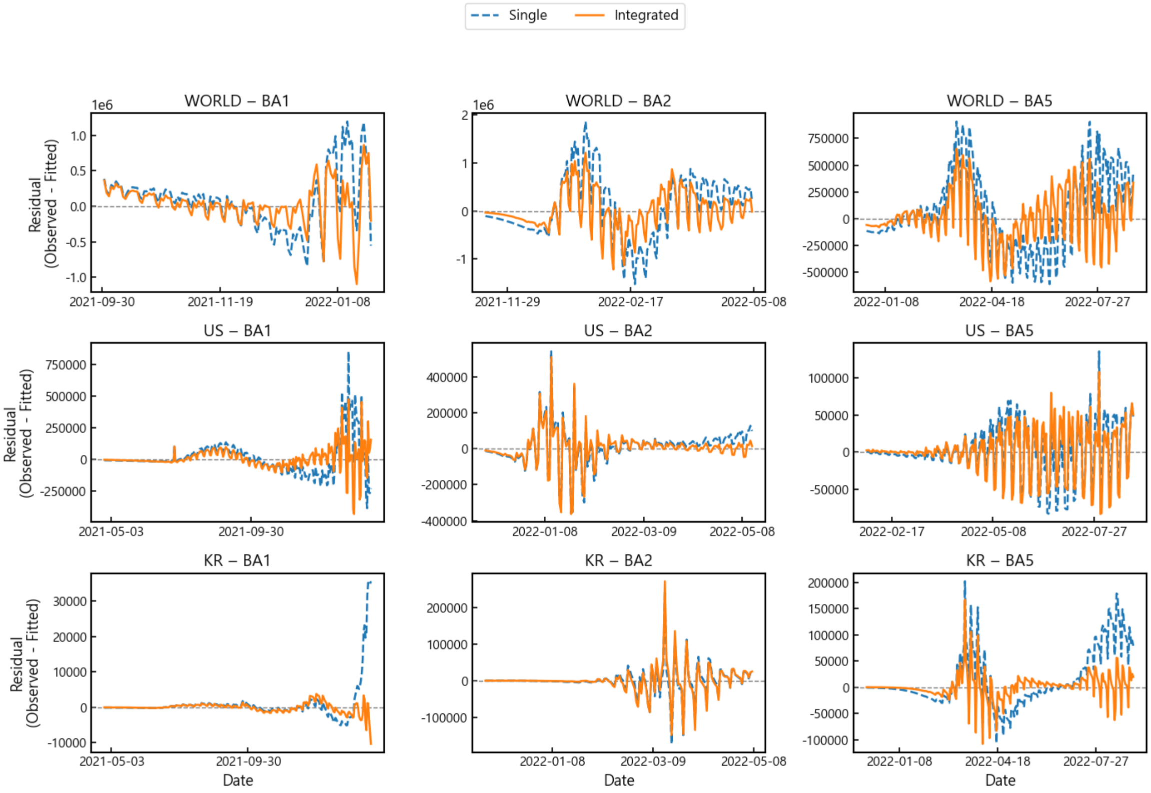

S3: Residual time series for single and integrated models in global total and the United States. Each panel shows residuals (observed minus fitted daily cases) for the single logistic model (dashed lines) and the integrated model (solid lines) across three variant transitions (Delta–BA.1, BA.1–BA.2, and BA.2–BA.5). Columns correspond to variant transitions and rows correspond to regions (World and USA). Residuals for the integrated model are generally smaller in magnitude and exhibit fewer systematic temporal patterns than those of the single model, supporting that the error reductions reported in the main text arise from better representation of multi-wave dynamics rather than overfitting random fluctuations.
