# Supplementary material for: Integrated disease model considering mutation-induced infection waves with COVID-19 cases
Source: PLoS One. 2026 Mar 6;21(3):e0341667. doi: 10.1371/journal.pone.0341667 (PMC12965675; doi:10.1371/journal.pone.0341667)
Supplement: S4 Text — MAPE, RMSE, and MAE values for the single model and integrated model across all study countries and variant transitions. (PDF) [file pone.0341667.s004.pdf]

Supporting Information
Integrated Disease Model Considering Mutation Induced Infection Waves with COVID-19 Cases

Seungho Baek *et al.*
Corresponding Author: Chansoo Kim, eau@ust.ac.kr.

S4. Comparison of error metrics across countries

S4: MAPE improvement (%p) by country and variant transition. Values represent the reduction in MAPE achieved by the integrated model compared to the single model. Positive values indicate the integrated model performs better.

**Country**	**Delta-BA.1**	**BA.1-BA.2**	**BA.2-BA.5**
World	12.89	19.84	25.46
Korea	21.41	3.51	42.24
USA	25.49	24.36	22.88
Japan	49.15	18.34	49.23
France	5.03	30.64	62.65
Australia	36.56	15.70	49.29
Canada	11.45	31.44	22.92
Israel	35.52	58.33	34.96
Chile	6.35	9.76	−1.43
Denmark	4.21	−0.01	14.72
Germany	−2.32	1.72	30.02
South Africa	1.51	27.79	−1.05
Singapore	0.63	0.59	13.26

Note: Negative values indicate cases where variant waves overlapped significantly within a short time interval, as discussed in the main text.
