# Supplementary material for: Integrated disease model considering mutation-induced infection waves with COVID-19 cases
Source: PLoS One. 2026 Mar 6;21(3):e0341667. doi: 10.1371/journal.pone.0341667 (PMC12965675; doi:10.1371/journal.pone.0341667)
Supplement: S5 Text — MAPE improvement (%p) for Chile, Denmark, Germany, South Africa, and Singapore across three major variant transitions (Delta–BA.1, BA.1–BA.2, BA.2–BA.5). (PDF) [file pone.0341667.s005.pdf]

Supporting Information

Integrated Disease Model Considering Mutation Induced Infection Waves with COVID-19 Cases

Seungho Baek *et al.*
Corresponding Author: Chansoo Kim, eau@ust.ac.kr.

S5. Error reduction rates for additional countries

S5: RMSE and MAE improvement by country and variant transition. Values represent the reduction in error (daily case counts) achieved by the integrated model compared to the single model. Positive values indicate the integrated model performs better.

	**Delta-BA.1**		**BA.1-BA.2**		**BA.2-BA.5**	
**Country**	RMSE	MAE	RMSE	MAE	RMSE	MAE
World	111,693	101,159	192,762	152,443	40,098	103,191
Korea	4,986	1,221	−1,503	−681	20,447	19,698
USA	47,539	29,810	6,065	10,073	59	1,104
Japan	15,692	5,766	7,504	6,503	14,523	11,880
France	3,380	1,188	45,867	42,777	2,625	15,824
Australia	3,962	2,387	4,440	3,404	1,699	3,347
Canada	3,106	1,705	2,689	2,632	−2	219
Israel	10,624	3,614	3,118	2,832	176	983
Chile	119	132	1,031	823	186	65
Denmark	454	154	389	352	56	231
Germany	5,050	1,364	9,076	7,336	13,162	22,315
South Africa	64	72	855	675	81	155
Singapore	17	4	3	12	565	556

Note: RMSE and MAE are absolute error metrics measured in daily case counts, which vary substantially across countries due to differences in population size and outbreak magnitude. MAPE (Table S1) provides scale-independent comparison.

To demonstrate the availability of generalization in the proposed integrated model, we evaluated its performance on 13 countries representing diverse geographic regions and epidemiological contexts. The integrated model outperformed the single model across all three metrics (MAPE, RMSE, MAE) in the majority of cases (35 of 39 country-transition combinations, 89.7%), with exceptions occurring primarily when variant waves overlapped significantly in time.
