# Supplementary material for: Integrated disease model considering mutation-induced infection waves with COVID-19 cases
Source: PLoS One. 2026 Mar 6;21(3):e0341667. doi: 10.1371/journal.pone.0341667 (PMC12965675; doi:10.1371/journal.pone.0341667)
Supplement: S6 Text — Detailed algorithm for applying PELT to averaged accuracy improvement curves. (PDF) [file pone.0341667.s006.pdf]

Supporting Information
Integrated Disease Model Considering Mutation Induced Infection Waves with COVID-19 Cases

Seungho Baek *et al.*
Corresponding Author: Chansoo Kim, eau@ust.ac.kr.

S6. Algorithm of Detection of Structural Transition in Accuracy Improvement

Algorithm: Detection of Structural Transition in Accuracy Improvement

Input:

- For each country or case $i = 1, \ldots, N$: time-series case data and variant share series $\phi \in [0, 100]\%$

Output: Set of change points $\{\phi_1^*, \phi_2^*, \ldots\}$ indicating structural shifts in accuracy gain

Step 1: Compute accuracy improvement per case
for each case $i = 1, \ldots, N$ **do**
  for each variant transition window **do**
    Fit a single model
    Fit integrated model (separated curves for dominant variant ranges)
    Compute $\text{MAPE}_{\text{single}}$ and $\text{MAPE}_{\text{multi}}$
    Compute $\Delta\alpha_i(\phi) = \text{MAPE}_{\text{single}} - \text{MAPE}_{\text{multi}}$
  end for
end for

Step 2: Interpolate each $\Delta\alpha_i(\phi)$ over a common grid $\phi = 0, 1, ..., 100\%$

Step 3: Compute the average accuracy improvement curve

$$\bar{\Delta}\alpha(\phi) = \frac{1}{N} \sum_{i=1}^{N} \Delta\alpha_i(\phi)$$

Step 4: Apply PELT to $\bar{\Delta}\alpha(\phi)$
Initialize cost function $\mathcal{C}(\cdot)$, penalty β, dynamic programming table F, and candidate set R
for $\phi = 1$ to 100 **do**
  Compute:

$$F(\phi) \leftarrow \min_{\phi' \in R} \left[F(\phi') + \mathcal{C}(\bar{\Delta}\alpha(\phi' + 1 : \phi)) + \beta \right]$$

  Update changepoint set $cp(\phi) \leftarrow cp(\phi^*) \cup \{\phi^*\}$
  Prune infeasible ϕ' and update candidate set
end for

return $cp(100)$ as final change points $\{\phi_1^*, \phi_2^*, \ldots\}$
